# Developing a Data-Driven Approach in Order to Improve the Safety and Quality of Patient Care

**DOI:** 10.3389/fpubh.2021.667819

**Published:** 2021-05-21

**Authors:** Fidelia Cascini, Federico Santaroni, Riccardo Lanzetti, Giovanna Failla, Andrea Gentili, Walter Ricciardi

**Affiliations:** ^1^Section of Hygiene and Public Health, Department of Life Sciences and Public Health, Università Cattolica del Sacro Cuore, Roma, Italy; ^2^Department of Statistical Sciences, Sapienza Università di Roma, Roma, Italy; ^3^Orthopaedics and Traumatology Unit, Department Emergency and Acceptance, San Camillo - Forlanini Hospital, Roma, Italy; ^4^Department of Public Health, University of Verona, Verona, Italy

**Keywords:** clinical governance, patient safety, guidelines, quality of care, best practices

## Abstract

**Objective:** To improve the safety and quality of patient care in hospitals by shaping clinical pathways throughout the patient journey.

**Study Setting:** A risk model designed for healthcare organizations in the context of the challenges arising from comorbidity and other treatment-related complexities.

**Study Design:** The core of the model is the patient and his intra-hospital journey, which is analyzed using a data-driven approach. The structure of a predictive model to support organizational and clinical decision-making activities is explained. Data relating to each step of the intra-hospital journey (from hospital admission to discharge) are extracted from clinical records.

**Principal Findings:** The proposed approach is feasible and can be used effectively to improve safety and quality. It enables the evaluation of clinical risks at each step of the patient journey.

**Conclusion:** Based on data from real cases, the model can record and calculate, over time, variables and behaviors that affect the safety and quality of healthcare organizations. This provides a greater understanding of healthcare processes and their complexity which can, in turn, advance research relating to clinical pathways and improve strategies adopted by organizations.

## Introduction

Healthcare firms have long identified the need to improve healthcare services, increasing quality and safety. Clinical pathways (CPs) were introduced for this purpose in the US in the early 1980s, being used as action strategies for specific patient groups (1). It was recommended that the development of CPs would be based as much as possible on scientific evidence for most common pathological conditions (2). The importance of managing patient risks properly was stated as of great importance (3).

In the past two decades, substantial efforts have been made in healthcare to improve quality of care and patient safety. Although improvements (i.e., development of patient-centered prototype) have been made, recent estimates continue to indicate the need for a marked change in approach (4, 5). Regarding quality and safety in particular, clinical pathways are considered as multidisciplinary and inter-professional plans that are related to specific categories of patients in precise local contexts, and their implementation has been evaluated according to process and outcome indicators (6–8). As a result, CPs are used as tools to facilitate and improve the delivery and the quality of healthcare services. Nonetheless, it has been noticed that in many healthcare settings, even developed ones, the use of them is not yet widespread (9).

The use of data-driven technologies is a promising opportunity for this purpose. Recent studies have already highlighted the importance and the necessity of developing a data-driven approach for the improvement in safety and quality of patient care (10, 11). Data-driven technologies operate through the collection, utilization, and analysis of patient data via the use of machine learning (ML) or other types of artificial intelligence (AI). They aid in harnessing and enhancing the breadth and depth of electronic health data to facilitate improved healthcare delivery for patients and the general public (12). Electronic medical records are included in the sources of data suitable for data-driven technologies.

Our model aims to improve the safety and quality of healthcare based on a data-driven approach. It has also been designed to have positive effects on the economy of healthcare providers because failure to properly consider and manage risk to patients has direct and indirect costs. Direct costs include: the costs of compensation and legal actions; the costs of additional treatments and prolonged hospital stays; refusals to reimburse certain services due to continual bad outcomes; losses from negative publicity or reputational damage. Indirect costs place a burden on society (i.e., increasing rate of morbidity, reduction of life expectancy).

In particular, this model makes use of a data-driven approach to improve patient outcomes and organizational efficiency by putting the patient and their intra-hospital journey at the center. The model can systematically keep track of all activities performed on a patient transversally to the healthcare organization, including all operational units and their structural, technological, and organizational aspects over time. It can reveal information and factors concerning adverse events and clinical complications, then analyze them to estimate the risk of expected, unexpected, or unwanted patient outcomes.

## Structuring a Data-Driven Approach

To construct a data-driven approach, it is necessary to proceed gradually through several steps. Below we suggest how to act.

### The Data Collection Procedure

The first and most fundamental part of the approach proposed in this study concerns data collection. The present model intends to consider the real intra-hospital journey experienced by the patient while receiving hospital care. As a result, we need a database that is represented by the clinical records (i.e., “the places” where the steps of the patient's hospital stay are all reported, in natural language) and includes sets of information such as physical examination on admission, courses, medication in progress, diagnosis of illnesses, laboratory tests or other tests results, surgical interventions, etc.

The patient's state at the time of discharge is treated as their “final” condition. This condition depends on the results of various steps during the intra-hospital journey, each being represented by a large amount of real-world data and collected in the form of medical records above described. Over time, the data gathered on the hospital-admitted patients becomes a rich source of available information to be used in the management of health risks and the improvement in the quality of healthcare services. Furthermore, such data can promote patient safety in conditions of complexity and on a systemic scale (i.e., on the level of the entire health organization).

The information in medical records (considered as elements of clinical events) cannot yet be used to manage clinical risk and improve the quality of healthcare organizations as a whole because they are written in indirect, natural language and not in the form of structured data. Automatic techniques that can transform medical records into structured information for algorithms applicable in the field of clinical governance (and the safety and quality areas in particular) are not yet available. This paper proposes how to manage unstructured data in order to make it available and useful to health professionals.

Automated data collection procedures can catalog not only the medical records but also organizational metadata, such as waiting times before clinical procedures, ward transfers, or the choice of room for specific treatment. Such metadata can also be used to define parameters in the process of the reconstruction and automatic evaluation of the intra-hospital journey. Once the structure that defines the parameters of the patient's intra-hospital journey has been created, these parameters can be standardized in the form of a data collection method that reveals patients' real journeys. After collection, data from medical records and metadata can be processed by *ad-hoc* algorithms to assess risks related explicitly to healthcare facilities over time.

### The Analytical-Descriptive Model

A model required to analyze patient outcomes and specific risks related to patient care in hospitals needs access to a significant collection of structured data that describes the real intra-hospital journeys of many patients, which has been collected in a standardized way as described in the previous section. With the aid of this model, the patient's journey can be seen as a path *P*, formalized as follows:

(1) $$\begin{array}{r} P=\;<I\to S1\to S2\to \ldots Sk\to > \end{array}$$

A path, *P*, starts from *I* and leads to *O*, passing through *k* intermediate steps, *Si*, where ϵ*{1,. k}*, sorted by time points. In particular, *I* = < *a, e, t, d, c, r>* and represents the initial condition of the patient characterized by: *a*- list of structured parameters that describe the patient's history information; *e*- list of structured parameters that describe the physical examination upon admission; *t*- list of structured parameters that describe the medications in progress upon admission; *d*- list of structured parameters that describe the diagnosis of the disease that makes hospitalization necessary; *c*- list of structured parameters that describe the complexity of the clinical case; *r*- list of structured parameters that describe the type of hospitalization; *o*- list of structured parameters that describe the expected clinical outcome. Table 1 shows some examples for each parameter.

**Table 1:** Example parameters describing the initial conditions of the patient.

*a*	List of structured parameters that describe the patient's history information: e.g., familiarity for pathologies, previous surgeries, allergies.
*e*	List of structured parameters that describe the physical examination upon admission: e.g., breath sounds, soft and non-tender abdomen, deep tendon reflexes.
*t*	List of structured parameters that describe the medications in progress upon admission: e.g., type and dosage of pharmaceutical drugs.
*d*	List of structured parameters that describe the diagnosis of the disease that makes hospitalization necessary: e.g., heart attack, stroke, pneumonia.
*c*	List of structured parameters that describe the complexity of the clinical case (diagnosis of all diseases present at admission: comorbidity factors, drug allergy, antibiotic resistance).
*r*	List of structured parameters that describe the type of hospitalization (emergency, elective, day hospital, or day surgery).
*o*	List of structured parameters that describe the expected clinical outcome (based on scientific evidence and clinical practice. They can be measured by activity data such as hospital re-admission rates, morbidity and mortality or by agreed scales).

Similarly, *S i* = < *s, p>* and represents a possible step or intermediate step on the patient's journey, characterized by a possible action or situation, *s*, that defines the transition from one step to the next, and by a set of structured parameters, *p*, that characterize the actions involving the patient or the situation the patient experiences. The final state, *O* = < *x,h>*, describes an expected or unintended outcome, *x*, and the associated health conditions, *h*, at the moment of discharge (including any complications).

**Figure 1:**
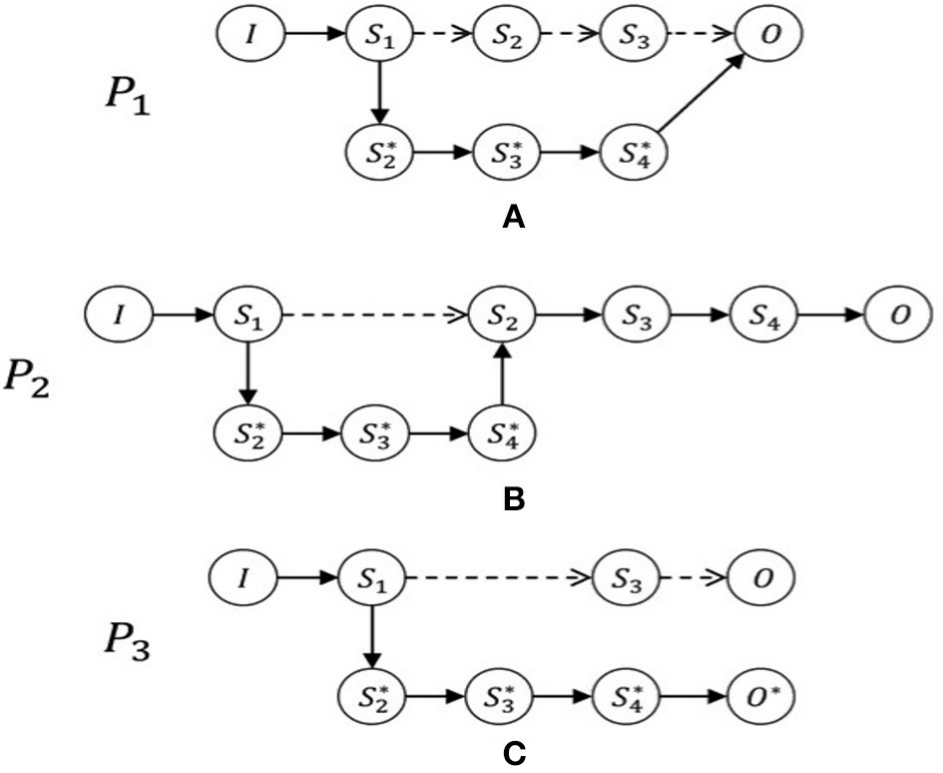
The patient journeys. Examples of journeys with possible variations: In **(A)**, journey *P1* varies from the initial expectations after step *S1*. It consequently then becomes the real journey, P1* and passes through new stages that had not been anticipated and finally arrives at the expected result, *O*. In contrast, journey *P2*, as shown in **(B)**, is varied by means of an additional stage between Steps *S1* and *S2*. In the journey *P3*, shown in **(C)**, we see a substantial variation from the expected journey, producing an unintended final condition, O* ≠ *O*. By analyzing the journeys of patients, it is possible to see how these are subject to changes, even when based on CPs. It is also possible to notice steps that patients have to pass through, interpret them, and consider the features of the variables depending on the healthcare facility and the health conditions of the particular patient.

Within this structure, we can model any patient's intra-hospital journey. In practice, once the initial condition has been established, the doctor plans the potential journey to reach the final condition on discharge (including compliance with guidelines, best practices, and evidence-based scientific knowledge) and identifies the expected outcomes. Therefore, upon hospital admission, the doctor will outline, at least at a conceptual or theoretical level, an expected journey *P* (see Figure 1 below). During the hospital stay, however, the expected journey could become real, as expected, or could vary at many points from the expectations. In the latter case, the model will define a new effective journey, P^*^, when a variation occurs, to confirm or deny the expectations of the doctor (Figure 1). Now we will present some case studies in order to provide practical examples of how the clinical path of a patient, although carefully designed in advance, can take alternative, unexpected paths. These real clinical cases regard patients admitted to the Emergency Department of the San Camillo Hospital in Rome (Italy) during 2018. Data were extracted from Electronic Health Records (EHR) of trauma patients and included: patient history, vital parameters, imaging, politrauma protocol, clinical examinations, laboratory analyses.

#### Case Study Description - 1

As a first example, we report the case of M.R., 40 years old, who was transported to the Emergency Room (ER) for a road traffic injury. Initial evaluation includes blood samples, politrauma diagnostic pathway with CT examination of head, thorax and abdomen, X-ray examination of the lower limbs. The initial diagnosis is a fracture of the distal femur (right side). The ER intervention is an application of damage control orthopedic protocol with external fixation of the injured limb, admission to the surgical ward for further clinical evaluation and diagnostic imaging and a CT examination of the operated limb for definitive surgical treatment planning.

Table 2 shows the description of process required in this case study.

**Table 2:** Information needed for the process of case study one.

	**Information**
1	Correct diagnosis and fracture pattern classification according to scientific knowledge and updated protocols
2	Preoperative planning according to the CT examination and fracture pattern: open reduction internal fixation with plate and screws
3	Surgical intervention with plate and screws of the distal femur (right side)
4	Full recovery of limb length, rotation, axis, and articular surface
5	Under-imaging with no CT scan evaluation of the fracture
6	Preoperative planning based only on X-ray examination and indication to internal fixation intramedullary nail
7	Surgical intervention with intramedullary nailing and screws of the articular surface and same outcome

The planned path would have been:

S1: correct diagnosis and fracture pattern classification according to scientific knowledge and updated protocols.
S2: preoperative planning according to the CT examination and fracture pattern: open reduction internal fixation with plate and screws.
S3: surgical intervention with plate and screws of the distal femur (right side).
S4: full recovery of limb length, rotation, axis, and articular surface.

Dismission.

But, unfortunately, some steps were different because of the hospital organizational failure:

S^*^2: under-imaging with no CT scan evaluation of the fracture.
S^*^3: preoperative planning based only on X-ray examination and indication to internal fixation with intramedullary nail.
S^*^4: surgical intervention with intramedullary nailing and screws of the articular surface and same outcome.

#### Case Study Description - 2

Another case study concerns A.V., 34 years old, who was transported to the ER for a road vehicle injury. Alcohol abuse has been reported and the patient presented the following values: GCS 15, FC 100 bpm, BP 140/90 mmHg, SpO_2_ 99%, EGA pH 7.3, PCO_2_ 43, PCO_2_ 101, HCO_3_−23, P/F 484, Lact 2.7, ethanol 2.54. From the ER evaluation, it was learned that GCS was 15, there was a preserved state of consciousness and there were no signs of neurological impairment, there was a local deformity on the lower limb (right side).

Table 3 shows the description of process required in case study two.

**Table 3:** Information needed for the process of case study two.

	**Information**
1	Polytrauma protocol (X-ray, CT total body, blood samples)
2	External fixation of the injured limb and CT examination for proper classification of the fracture pattern
3	Definitive fixation with full recovery
4	Incorrect preoperative planning with no CT examination and improper internal fixation of the fracture
5	Malunion of the fracture
6	Bone osteosynthesis review with bone deformity correction and partial articular recovery of the ankle joint

The following series of events should have happened:

S1: polytrauma protocol (Xray, CT total body, blood samples)
S2: external fixation of the injured limb and CT examination for proper classification of the fracture pattern
S3: definitive fixation with full recovery

However, some steps were different from what had previously been planned because of the hospital organizational failure, and in particular:

S2^*^ incorrect preoperative planning with no CT examination and improper internal fixation of the fracture
S3^*^ malunion of the fracture
S4^*^ bone osteosynthesis review with bone deformity correction and partial articular recovery of the ankle joint.

### The Predictive Model

To build a predictive model of patient health risk, we must analyze different cases. So, we have imagined two different scenarios that could help us in the realization of the predictive model: a closed world hypothesis and a real context. This will provide an understanding of the methodologies, challenges and opportunities that these solutions offer.

#### Scenario 1 - Closed World Hypothesis

**Figure 2:**
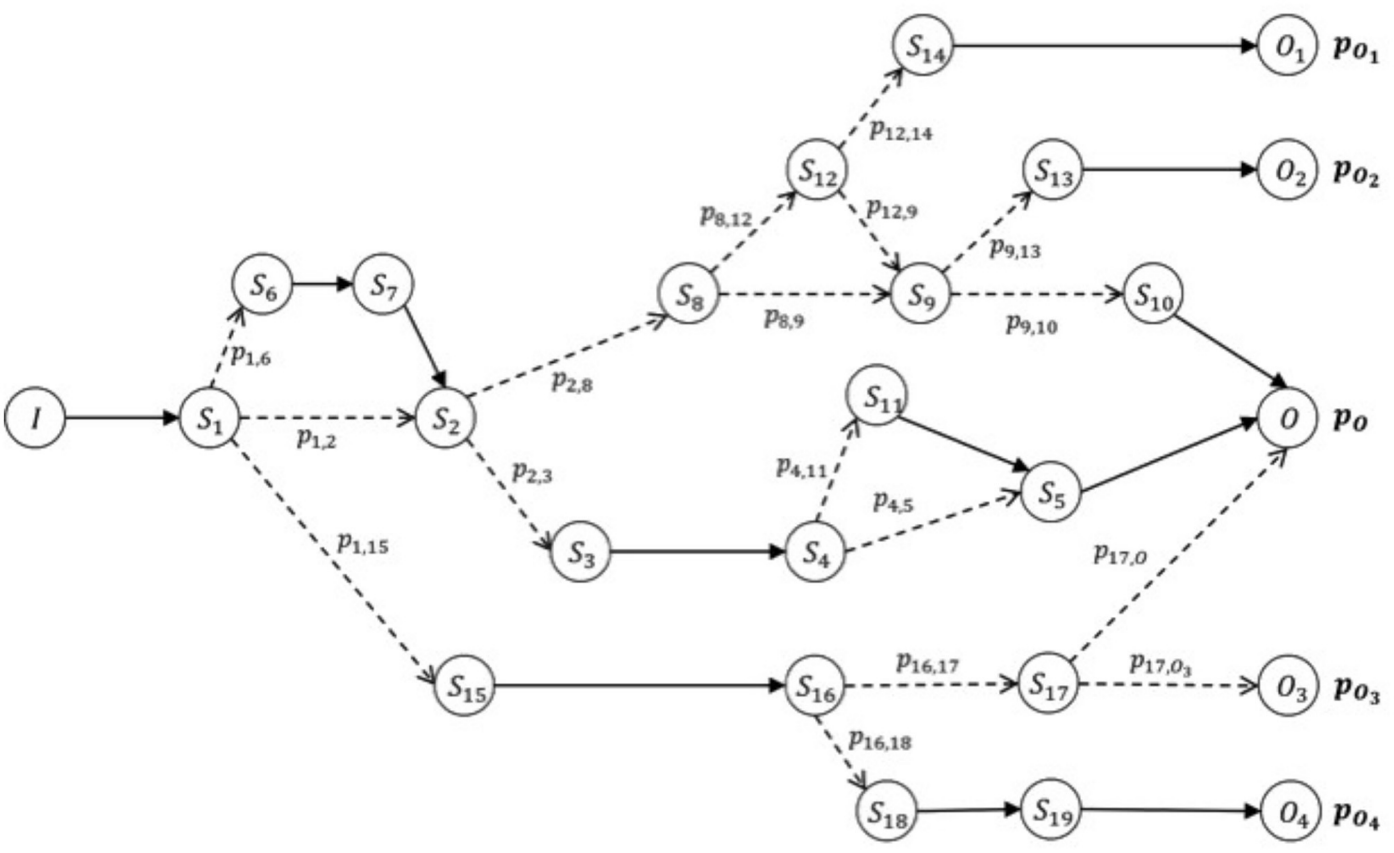
Oriented graph of possible patient journeys. Example of a graph that models the data collected for a cluster of journeys, according to their specific initial features (condition on admission), *I*. The expected (or positive) result of this path is *O*, which can be reached with probability *pO*. The patient's risk is defined by the probability of reaching different final conditions given the same starting condition upon admission. The risk of obtaining a final condition different from *O*, starting from condition *I* on admission, is given by the list of probabilities *[p (O1), p (O2), p (O3), p (O4)]*. Each node of the graph represents a possible step (initial, intermediate, or final) of the journey (and thus a stage of the patient's condition between admission and discharge), and each edge (or line between nodes) represents a possible succession of steps/stages according to the probability of encountering each of them. Such journeys make up our knowledge base. In particular, we can calculate the probability of arriving at the final step/stage, given any initial node of the graph, be it initial or intermediate.

The hypothesis of a closed world is a theoretical simplification for which we hypothesize our domain as completely represented by the clinical cases collected (clinical cases that have never been observed are assumed to be non-existent). We will then consider the hypothesis in which, for a specific healthcare facility, the available databases cover, over time, a sufficient amount of data to include all possible patient journeys. In this case, the amount of data is capable of being handled easily by the healthcare facility and has sufficient statistical significance. In this scenario, we can group the journeys into clusters according to the parameters of the initial condition (on admission) such as diagnosis and comorbidity of the patient. We can then go on to obtain, for each of these groups (or clusters), a directed graph that shows all types of the journey, from the initial condition to various final conditions, through different possible stages (Figure 2).

This model defines a measure of health risk as the probability of reaching a different final step/stage, *Oi*, compared to the expected one *O*. At the time of the patient's admission (when we define the initial condition of his real journey), we identify the cluster to which the patient belongs. Then we interrogate the associated graph to determine what the expected risk is, and the probability that this patient will find himself at the end of his intra-hospital journey in a final condition different from the expected one upon admission. By using this information, physicians can make informed choices that help them implement clinical protocols and best practices according to real patients' situations.

A potential extension of this mode includes the possibility to show the probabilities related to different steps the current patient journey may lead to in advance. This can provide useful information to reinforce the choices made by professionals. In certain conditions (depending on the type of clinical journey that is taking shape), the model may highlight not only the risk variations according to the patient final condition or the final stage of the intra-hospital journey, but also what the probabilities are to reach a subsequent intermediate stage. Example: a patient is at Stage A; many important things could happen here that could lead to Stages B, C or D after A, and the model could suggest that there is a 65% probability of ending up in Stage B, 25% in Stage C, and 10% in Stage D. Importantly, when we store a new intra-hospital journey in line with Scenario 1, the new journey can be added to the existing knowledge base using appropriate methods. This helps to guarantee the validity of the operation and to increase the accuracy of the model.

#### Scenario 2 - Real Context

In this scenario, we cannot assume that we have data on all possible combinations of clinical cases that a healthcare facility may deal with. This is due to the intrinsic difficulty of gathering such a large quantity of information in the real world and because in the field of medical science, illnesses, technologies and therapies are continuously evolving and constantly present new information. Unlike in Scenario 1, we cannot make simplistic assumptions about our domain.

Furthermore, in the absence of structured data collected with a methodology that follows precise criteria, it would not be likely the model could assess the data domain with the right degree of detail. Nor would it be possible to verify the accuracy, effectiveness or reliability of specific hypotheses that support the implemented algorithms.

However, the representation of our knowledge base can be used as a starting point to define approaches based on machine learning algorithms that are able to give us a reliable prediction of previously defined health risks. These approaches may involve the use of techniques with different levels of complexity (i.e., decision trees, random forests, support vector machines and even advanced deep learning techniques).

Regardless of the type of algorithmic approach that would be the most appropriate for the collected knowledge base, a risk prediction can be obtained both as a function of the initial situation on admission and in progress (step-by-step) during the intra-hospital patient journey. Given the patient's initial condition, the model can estimate the health risk and, similarly, the risk variation when the patient passes to a new stage (possibly, but not necessarily, using different algorithmic techniques).

## Customizing the Model with Hospital-Related Variables

We have considered a model that could describe the real intra-hospital journey experienced by the patient and have aimed to identify deviations from expected CPs. By using this model, we can calculate the risks to a patient while receiving hospital care as well as estimate conditions on discharge and at intermediate stages in comparison to those upon admission. Variables related to a specific healthcare facility, which can influence the patient conditions during the intra-hospital journey, are implicitly taken into account when we collect data relating to a single hospital. This means considering the risk to the patient as measured at the start of the journey and at intermediate stages on which the knowledge base has been built.

We might further increase the capacity and the precision of the model based on a data-driven approach if we consider specific factors that characterize each healthcare facility that influences patient journeys. They can be summarized as:

- The size (number of beds) and the number of specialist departments.
- The type of organization (specialty clinics, University hospital, etc.).
- The clinical cases and their statistics.
- The personnel responsible for the patient.

Assuming this information can be structured in such a way as to be processed by algorithmic models, these parameters should characterize not a single patient journey but, of course, all intra-hospital journeys.

## Discussion

Clinical pathways are useful for planning care processes, implementing clinical governance policies, rationalizing health service provisions, improving the quality of care and outcomes, reducing risks and medical errors, and increasing patient satisfaction (13–15). At the same time, CPs must also be kept up-to-date and reflect evidence-based medicine, as outlined in the latest guidelines. However, it has been noted that there are different terms for “clinical pathways” in various studies and a standard definition is still lacking (14–16). This inconsistency has unfavorable effects on the coherent transmission of scientific evidence of health care practices as well as on the design and implementation of CPs themselves. To overcome the problem of clinical pathways taxonomy, attempts to describe them and their impact on hospital governance have been made (16) and a minimum criterion has been identified (9, 15, 17–19).

Physicians could use clinical decision support systems (CDSS) with Big Data analytics to make more informed decisions, which may improve the quality of patient care. The first data-driven clinical decision-making and hospital information system (HIS) is named the HELP (Health Evaluation via Logical Processing). The HELP system is comprised of a knowledge base, data, a decision-making processor, data review, time driver, patient database and accounting system (11). The system utilizes its knowledge base to organize all of the multifaceted information. The manual “Crossing the Quality Chasm: A New Health System for the 21st Century” (4) states the following about CPs: “For some conditions, a set of clearly identified processes should occur. In complex adaptive systems such as health care, however, few patient care processes are linear.” In respect to healthcare organizations in particular, coordination requires the design of procedures that are responsive to unexpected occurrences. Previous research has been performed regarding this line of work and has enhanced the importance of clinical pathways to be based on real data, using information extracted from Electronic Patient Records where possible (20).

Different clinical pathways approaches are proven to support the decision-making process of healthcare professionals. In terms of treatment decisions, it has been proposed to support resolutions made by family physicians on the management of patients with atrial fibrillation and chronic heart failure. This has been done by the translation of guidelines into disease-specific CPs that consider specialized comorbidity management procedures resulting in the development of a unified model of knowledge (21). Concerning the aim of increasing hospital efficiency by developing a decision support tool, a simulation-optimization model has been used in surgery for the optimization of hospital resources such as ward beds and operating rooms. (22).

Data-driven approaches can be applied to CPs to manage clinical risks more appropriately, consequently improving safety and quality of healthcare organizations in the perspective of a digitized smart hospital. They can help healthcare professionals to decide on the best care strategy, to facilitate the correct management interventions and to result in a reduced length of hospital stay (23). However, to support this standardized and systematic data collection process operatively, this data-driven approach requires a stand-alone information system or an extension of systems possibly already in use in the healthcare facility. Both options require a developmental phase before implementation, involving multidisciplinary cooperation between experts in medicine, healthcare, data science and information systems. The ultimate outcome of the proposed model is information that can be used to shape CPs by outlining patients' health risks (and other undesired outcomes) that are related to their real intra-hospital journeys.

## Conclusion

In conclusion, this paper proposes a model which can positively influence both the treatment decisions as well as organizational efficiency. It is based on real journeys of patients during hospital stays and makes use of data extracted from clinical records, from admission through discharge and all of the intermediate steps along the way. By tracking changes in the constitutive elements of a healthcare organization, including the activities of all health professionals, the data-driven approach proposed in this paper can be applied as a reference to compare the quality and safety of different healthcare facilities and the measures that may need to be taken to comply with accreditation requirements for healthcare organizations.

## Data Availability Statement

The original contributions presented in the study are included in the article/supplementary material, further inquiries can be directed to the corresponding author.

## Author Contributions

FC and FS conceived the study. RL, GF, and AG designed the journey real examples. FC and WR wrote the manuscript with the inputs from all the team. All authors contributed to the refinement of the study and approved finally the manuscript.

## Conflict of Interest

The authors declare that the research was conducted in the absence of any commercial or financial relationships that could be construed as a potential conflict of interest.
